# Photosynthetic poly-β-hydroxybutyrate accumulation in unicellular cyanobacterium *Synechocystis* sp. PCC 6714

**DOI:** 10.1186/s13568-017-0443-9

**Published:** 2017-07-06

**Authors:** Donya Kamravamanesh, Stefan Pflügl, Winfried Nischkauer, Andreas Limbeck, Maximilian Lackner, Christoph Herwig

**Affiliations:** 1 Institute of Chemical, Environmental and Biological Engineering, Research Area Biochemical Engineering, Technische Universität Wien, Gumpendorfer Straße 1a, 1060 Vienna, Austria; 2 Institute of Chemical Technologies and Analytics, Technische Universität Wien, Vienna, Austria; 3 Lackner Ventures & Consulting GmbH, 1210 Vienna, Austria; 4 University of Applied Sciences FH Technikum Wien, Hoechstaedtplatz 6, 1200 Vienna, Austria

**Keywords:** Photobioreactor cultivations, Cyanobacterium, Poly-β-hydroxybutyrate, *Synechocystis* sp. PCC 6714, Nitrogen and phosphorus limitation

## Abstract

Poly-β-hydroxybutyrate (PHB) production from CO_2_ has the potential to reduce the production cost of this biodegradable polyesters, and also to make the material more sustainable compared to utilization of sugar feedstocks. In this study the unicellular cyanobacterium, *Synechocystis* sp. PCC 6714 has been identified as an unexplored potential organism for production of PHB. *Synechocystis* sp. PCC 6714 was studied under various cultivation conditions and nutritional limitations. Combined effects of nitrogen and phosphorus deficiency led to highest PHB accumulation under photoautotrophic conditions. Multivariate experimental design and quantitative bioprocess development methodologies were used to identify the key cultivation parameters for PHB accumulation. Biomass growth and PHB accumulation were studied under controlled defined conditions in a lab-scale photobioreactor. Specific growth rates were fourfold higher in photobioreactor experiments when cultivation conditions were controlled. After 14 days of cultivation in nitrogen and phosphorus, limited media intracellular PHB levels reached up to 16.4% from CO_2_. The highest volumetric production rate of PHB was 59 ± 6 mg L^−1^ day^−1^. Scanning electron microscopy of isolated PHB granules of *Synechocystis* sp. PCC 6714 cultivated under nitrogen and phosphorus limitations showed an average diameter of 0.7 µm. The results of this study might contribute towards a better understanding of photoautotrophic PHB production from cyanobacteria.

## Introduction

Today, petroleum-based plastics are an essential part across all industries and have replaced glass and paper in packaging (Khanna and Srivastava 2005). Global plastics production reached around 322 million tonnes in 2015 (Plastics Europe 2016). Accumulation of these non-biodegradable plastics in the environment is a worldwide concern (Thompson et al. 2009), e.g. as microplastics in the marine environment. In this context, attention has been focused on research for the production of biodegradable plastics (Samantaray and Mallick 2015). Polyhydroxybutyrate (PHB) is the best-characterized member of the polyhydroxyalkanoate (PHA) family and is widespread in various bacterial species as storage material (Liebergesell et al. 1994; Steinbüchel 2008).

PHB is biodegradable (and compostable according to EN 13432), insoluble in water, non-toxic and biocompatible. Therefore, PHB could be an attractive alternative to petroleum-based plastics (Samantaray and Mallick 2015). It resembles the commodity polymer polypropylene in its properties (Lackner 2015). PHB is commercially produced by heterotrophic bacteria such as *Alcaligenes eutrophus* (Madison and Huisman 1999), *Alcaligenes latus* (Grothe and Chisti 2000) and recombinant *Escherichia coli* (Schubert et al. 1988). Despite relatively high yields of PHB, production from bacterial fermentation requires sugar supplementation and continuous oxygen supply which results in high substrate and operation costs (Steinbüchel 2008; Wu et al. 2001). In addition, public discussion about bioplastics production from sugar feedstocks is similar to the discussion about first generation biofuels. Competition of material with food and feed production for the same resources potentially leads to shortages and price increases causing poverty and is also contributing to climate change through direct and indirect land use change (Chen et al. 2017).

In order to compete with common plastics, cost reduction in PHB production is obligatory. This could possibly be achieved using cheap substrates such as whey, hemicellulose, and molasses (Alias and Tan 2005; Reddy et al. 2003). In this context, cyanobacteria are emerging as an alternative host system due to their minimal nutrient requirements and photoautotrophic nature (Samantaray and Mallick 2012). To date, PHB accumulation has been reported for a few cyanobacterial species with photoautotrophic PHB production reaching less than 10% of dry cell weight (dcw) (Bhati et al. 2010). The thermophilic cyanobacterial strain *Synechocystis* sp. MA-19 is the sole exception with accumulation of 27% (dcw) PHB reported (Miyake et al. 2000). A higher PHB content in cyanobacteria has only been detected in the case of heterotrophy or genetic modification of the strain; 29 and 41.6% (dcw) PHB were obtained in the presence of acetate and under P-deficiency in *Synechocystis* sp. PCC 6803 and *Nostoc muscorum*, respectively (Panda et al. 2006; Sharma et al. 2007).

The aim of this study was to quantitatively investigate the unicellular non-nitrogen fixing cyanobacterial strain *Synechocystis* sp. PCC 6714 for its ability to produce PHB. This strain has not been studied yet for PHB production but its recently published genome sequence (Kopf et al. 2014) showed a close relation to the widely studied model organism *Synechocystis* sp. PCC 6803 which has been extensively studied for PHB accumulation. Hence, the potential of PHB accumulation in *Synechocystis* sp. PCC 6714 was evaluated by shake flask experiments as well as bioreactor cultivations under defined conditions and the volumetric rates and productivities were evaluated. Additionally, growth behavior on different carbon sources as well as different cultivation conditions and their effect on biomass and PHB formation were investigated. Finally, the morphology of PHB granules has been studied using scanning electron microscopy (SEM). Advanced quantitative process development approaches including multivariate experimental design were used, aiming to provide a quantitative and consistent data-set to the scientific community.

## Materials and methods

### Strain and growth conditions

An axenic culture of wild-type strain *Synechocystis* sp. PCC 6714 was purchased from Pasteur Culture Collection of Cyanobacteria (Pasteur Institute, Paris, France). Unless stated otherwise, *Synechocystis* sp. PCC 6714 was grown in BG-11 medium (Rippka et al. 1979) supplemented with 10 mM HEPES buffer pH 8.5 and 5 mM NaHCO_3_ as carbon source prior to inoculation. In order to induce nitrogen deficiency, cells were cultured in BG-11 media without nitrate and ammonia. Ferrous ammonium citrate and Co (NO_3_)_2_·6H_2_O were substituted with equimolar concentrations of Ferric citrate and CoCl_2_·6H_2_O in terms of iron and copper content. For phosphorus limitation, KH_2_PO_4_ was replaced with an equimolar concentration of KCl in terms of potassium content (Panda et al. 2006).

The impact of acetate supplementation on PHB accumulation was studied by taking cells from their late exponential growth phase and transferring them into media without nitrogen or without nitrogen and phosphorus source and 5 mM acetate as substrate. PHB content was analyzed on 3rd, 7th, 14th and 16th day of incubation.

Cultivations were done in 500 mL Erlenmeyer flasks containing 100 mL medium at 28 ± 2 °C under continuous illumination with 50 ± 5 µmol photon m^−2^ s^−1^ in photosynthetically active radiation (PAR) in a shaking incubator (Infors, Switzerland) at 100 rpm agitation.

The impact of different carbon sources on biomass growth was studied in shake flask experiments under continuous illumination using BG-11 media and 5 mM of either carbonate, acetate, glucose or glycerol.

### Growth determination and estimation of dry weight

Biomass growth was determined spectrophotometrically at 750 nm using a UV–Vis Spectrophotometer (Thermo Scientific, USA) at 24-h intervals. Dry Cell Weight (dcw) was determined in triplicates by transferring 10 mL of fermentation broth or shake flask cultures to a reusable pressure filter holder (Sartorius, Göttingen, Germany) and by filtering on a pre-weighed 0.45 µm Cellulose acetate filter paper (Sartorius, Göttingen, Germany) at a pressure of 6 bars for 1 min. Filters were dried overnight at 60 °C and dry weight was determined gravimetrically. A correlation between optical density and dcw could be established and described in Eq. (1)

1 $$ {\text{C}}_{\text{x}} =  0.425{\text{ OD}}_{750} . $$

### Gas-exchange limitation or heterotrophy and nutrient limitations

Impact of gas exchange limitation (i.e. limitation of gas transfer to the culture vessel) was studied using cultivations on media without nitrogen or without nitrogen and phosphorus source in 150 mL anaerobic flasks plugged with rubber stoppers under three different illumination conditions, dark, light and dark/light cycle (16:8) h, respectively.

### Design of experiments

Full factorial experimental design with three center points and data evaluation were carried out using MODDE (Umetrics, Sweden). For each selected response, MODDE generates a multiple linear regression (MLR) model with key parameters of R^2^ (coefficient of determination), Q^2^ (predictability), RP (reproducibility) and MV (model validity). The thresholds for R^2^, Q^2^, and RP were 0.5 for each parameter. Models with an MV value of >0.25 were considered to be significant.

### Bioreactor cultivations

Bioreactor experiments were carried out under sterile conditions in a 1.5 L jacketed glass reactor with a working volume of 1 L (Applikon B.V, the Netherlands). The temperature was maintained at 28 °C and pH was measured with a pH-electrode (Mettler Toledo GmbH, Vienna, Austria) and was automatically maintained at 8.5 by the addition of 0.5 M HCl or 0.5 M NaOH. Agitation speed was at 300 rpm. Gas flow was controlled by mass flow controllers for air and CO_2_ (Brooks Instrument, Matfiels, USA). The reactor was bubbled with a mixture of sterile filtered air and 2% CO_2_ at a flow rate of 0.02 vvm (20 mL min^−1^). The illumination was done using LED strips wrapped around the reactor vessel providing a light intensity of 40 µmol m^−2^ s^−1^ photons in PAR. O_2_ and CO_2_ were measured in the off-gas with BlueSens gas sensors (BlueSens GmbH, Herten, Germany).

All fermentation parameters and variable pump set-points were controlled using the process information management system Lucullus online monitoring system 3.2 (Securecell AG, Schlieren, Switzerland).

Photobioreactor experiments were performed in duplicates and one is shown as an example. Samples were taken in triplicates at 24-h intervals and were analyzed for biomass, PHB content, and concentrations of macronutrients.

### Determination of the PHB content

PHB quantification was done using the procedure described by Schlebusch and Forchhammer (2010) and Taroncher-Oldenburg et al. (2000). Pre-weighed dried cells (5–10 mg) were boiled with 1 mL conc. H_2_SO_4_ at 100 °C on a heating block (Accublock™, Labnet, USA) for one hour to convert PHB to crotonic acid. Samples were allowed to cool down and subsequently diluted 20 times using 0.014 M H_2_SO_4_. Crotonic acid was determined using a high-performance liquid chromatography system (Thermo-Fischer Scientific, USA) with a Nucleosil C8 column (Macherey–Nagel, Germany) using an isocratic method. The mobile phase used was 20 mM NaH_2_PO_4_ buffer; pH 2.5 and acetonitrile (70:30) with a flow rate of 0.85 mL min^−1^ and a column temperature of 30 °C. Detection of crotonic acid was done using a diode array detector (DAD) detector (Thermo-Fischer Scientific, USA) at 210 nm. For calibration, pure PHB (Sigma-Aldrich, USA) was treated accordingly and analyzed in parallel with samples. Instrument control and peak evaluation were done with Chromeleon 7.2 (Thermo-Fischer Scientific, USA). The percentage (dcw) PHB was determined with the amount of PHB obtained from HPLC analysis and the cell dry weight of biomass used for the analysis using Eq. (2):

2 $$ \% \;{\text{dcw}}\;PHB = \frac{{mg ({\text{PHB}})}}{mg (dcw)}*100. $$

### Analysis of micronutrients

The concentrations of nitrogen (N), phosphorus (P), sulfur (S), and carbon (C) were measured using inductively coupled plasma-optical emission spectroscopy (ICP-OES) according to the modified method described previously by Nischkauer et al. (2014). For the analysis, samples were diluted (1:2) with 1% (v/v) hydrochloric acid and iridium was added as internal standard (final concentration 5 µg mL^−1^). Calibration solutions were prepared from the analytical grade salts of (sodium dihydrogen phosphate dihydrate for P, potassium nitrate for N, sodium sulfate for S, and sodium acetate for C). Samples and standards were analyzed with an iCAP 6000 ICP-OES instrument (Thermo Scientific, Germany). The optimized ICP parameters are given in Table 1. For each element three replicates were measured, two emission lines were monitored (for nitrogen, only one suitable line was available in the investigated spectral range), and quantitative results were calculated from both emission lines. The counts observed were converted into concentration units by means of external aqueous calibration. The response of the internal standard (Ir) was constant over each measurement session (5% relative standard deviation, no temporal trend), and no difference in Ir-response between samples and calibration standards was observed.

**Table 1:** Represents the optimized ICP parameters used for determination of nitrogen, phosphorus, sulfur and carbon

Exposure time	7 s
RF power	1400 W
Nebulizer gas flow	0.8 L min^−1^ argon
Viewing height above load-coil	11 mm
Cooling gas flow	15 L min^−1^ argon
Auxiliary gas flow	2 L min^−1^ argon
N 174.272 nm	^a^
P 213.618 nm	P 178.284 nm
S 182.034 nm	S 180.731 nm
C 175.183 nm	C 193.091 nm

*RF* radio frequency

^a^N has only one useful emission line in the spectral range investigated

### Analysis of PHB granules using scanning electron microscopy (SEM)

50 mL cyanobacterial cell suspension containing PHB granules was homogenized using Panda Plus homogenizer (GEA Group AG, Düsseldorf, Germany) for ten passages at 1500 bar pressure at room temperature. The homogenate was centrifuged at 10,000 rpm at 4 °C for 10 min. In order to remove pigments, the pellet containing PHB granules was washed with methanol. The cell pellet was subsequently harvested by centrifugation at 3000 rpm and resuspended in ultrapure water. 100 µL of an appropriate dilution of the suspension was transferred on a gold-sputtered (10–50 nm) polycarbonate filter (Millipore-Merck, Darmstadt, Germany) using reusable syringe filter holders with a diameter of 13 mm (Sartorius, Göttingen, Germany) and filtered using pressurized air. Gold-sputtered filters were washed with 200 µL of ultrapure water and fixed on a SEM sample holder using graphite adhesive tape and subsequently sputtered with gold to increase the contrast. SEM was performed using a QUANTA FEI SEM (Thermo Fisher, Waltham, MA, USA) with a secondary electron detector (SED). The acceleration voltage of the electron beam was set between 3 and 5 kV. The diameter of the PHB granules on SEM pictures was evaluated using the ImageJ plugin Fiji (Laboratory for Optical and Computational Instrumentation (LOCI), University of Wisconsin-Madison, WI, USA).

## Results

### Strain characterization

#### Impact of carbon sources on biomass formation

Initial characterization by shake flask experiments was done to gain a better understanding of cyanobacterial strain *Synechocystis* sp. PCC 6714. Different carbon sources, namely carbonate, acetate, glucose and glycerol were studied with respect to their impact on biomass growth.

**Fig. 1:**
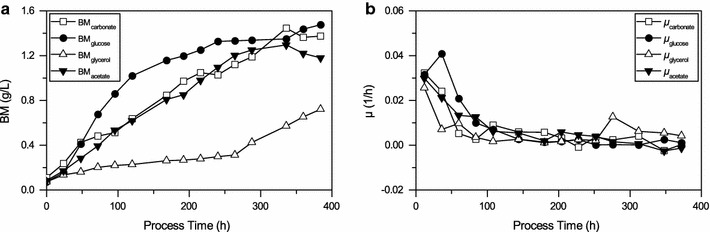
**a** Growth curves and **b** average specific growth rates of *Synechocystis* sp. PCC 6714 on 5 mM carbonate, acetate, glucose, and glycerol (*BM* biomass). The values shown are the mean of three individual experiments, a single standard deviation is given as *error bar*

As shown in Fig. 1 growth on carbonate reached a maximum biomass concentration of 1.4 ± 0.15 g L^−1^ after 14 days of incubation with an average specific growth rate (μ_average_) of 0.095 ± 0.01 day^−1^ and a maximum specific growth rate (μ_max_) of 0.225 ± 0.02 day^−1^. Growth on acetate reached a maximum biomass concentration of 1.3 g L^−1^ after 14 days of cultivation with a μ_average_ of 0.09 ± 0.01 day^−1^ and a μ_max_ of 0.2 ± 0.02 day^−1^, which are slightly lower when compared to growth on carbonate. Growth on glucose occurred with a μ_average_ of 0.107 ± 0.01 day^−1^ and a μ_max_ of 0.28 ± 0.02 day^−1^ which is higher when compared to growth on carbonate and acetate. Growth on glycerol occurred with a lag phase of 250 h. After that, a μ_average_ of 0.09 ± 0.01 day^−1^ a biomass concentration of 0.72 ± 0.1 g L^−1^ was observed. This value was comparable with cultivations on carbonate and acetate. The results showed that *Synechocystis* sp. PCC 6714 can grow under different conditions ranging from fully autotrophic to heterotrophic growth conditions.

#### PHB formation in *Synechocystis* sp. PCC 6714

In order to study whether *Synechocystis* sp. PCC 6714 is able to accumulate PHB, cultivations under various nutrient limitations and continuous illumination were performed. The impact of the nitrogen source during growth (nitrate or ammonia), gas exchange limitation (GEL) and heterotrophy were studied under nutrient limitation. Experiments were carried out in two steps. After the growth phase on complete BG-11 media using nitrate or ammonia as a nitrogen source, limiting conditions were achieved by transferring cells to media without a nitrogen or without a nitrogen and phosphorus source, while using carbonate or acetate as substrate. Table 2 shows the PHB content obtained for *Synechocystis* sp. PCC 6714 under various limiting conditions. 7.5 ± 1% (dcw) PHB was obtained after 7 days of nitrogen limitation for cells previously grown on nitrate. This amount was further increased to 10 ± 1% (dcw) when cultivation was prolonged to 14 days. PHB content was higher when compared to cells previously grown on ammonia, where 9 ± 1% (dcw) were obtained after 14 days of limitation. After 16 days of nitrogen limitation in case biomass was grown on nitrate and acetate was the supplemented carbon source, 14.9 ± 1% (dcw) PHB was obtained. After 7 days of cultivation under nitrogen and phosphorus limitation, 8.2 ± 1% (dcw) PHB accumulation was detected and the highest PHB content of 13 ± 1% (dcw) was found after 14 days of limitation. The intracellular PHB level reached 15.5 ± 2% (dcw) after 16 days of incubation under nitrate and phosphate starvation when 5 mM acetate was supplemented. The PHB accumulation was higher for both nitrogen and nitrogen and phosphorus limitation when nitrate was used as nitrogen source. Gas exchange limitation under nitrogen and phosphorus limitation was done in order to study the effect of preventing an exchange of gas between the culture vessel and the environment on PHB accumulation. As shown in Table 2, a significant reduction in the PHB pool of *Synechocystis* sp. PCC 6714 was observed when the transfer of gas into the culture vessel was limited. The PHB content obtained was only 2.2 ± 1% (dcw) after 7 days of limitation and this amount was even further reduced when cultivation was prolonged to 14 days. Higher biomass concentrations were observed under nitrogen limitation than under nitrogen and phosphorus limitation. In addition, an insignificant increase in biomass concentration under GEL was observed.

**Table 2:** PHB concentrations in *Synechocystis* sp. PCC 6714 grown under continuous illumination and different limiting conditions

Limitation condition	3 day	7 day	14 day	16 day
Cells growing of complete BG-11 media	0	<0.5%	<1%	1%
Nitrogen limitation (growth on nitrate), %	2.5	7.5	10	9.5
Nitrogen limitation (growth on ammonia), %	2	6	9	8.5
Nitrogen limitation (growth on nitrate) and 5 mM acetate, %	3	7.9	11.4	14.9
Nitrogen limitation (growth on ammonia) and 5 mM acetate, %	3	7.2	11	14
Nitrogen and phosphorus limitation (growth on nitrate), %	3.4	8.2	13	11
Nitrogen and phosphorus limitation (growth on ammonia), %	2.9	7	12.5	12
Nitrogen and phosphorus limitation and 5 mM acetate, %	3.2	7	12.4	15.5
Nitrogen, phosphorus and gas exchange limitation, %	<1	2.2	1.5	1

The values shown are the mean of three individual experiments

### Influence of cultivation conditions on PHB accumulation using a multivariate design of experiments (DoE)

In this study, the ability of *Synechocystis* sp. PCC 6714 to accumulate PHB was elucidated. The highest photoautotrophic PHB content of 13 ± 1% (dcw) was obtained under nitrogen and phosphorus limitation. To better understand the capability of the strain to produce biomass and PHB, the influence of temperature, pH, and CO_2_ availability was investigated. To that end, a full factorial design of experiments under nitrogen and phosphorus limitations was carried out. The experiments and the parameters are given in Table 3. It was hypothesized that PHB accumulation in strain *Synechocystis* sp. PCC 6714 is influenced by cultivation conditions. The two responses, biomass growth, and PHB accumulation after 14 days were determined and the results of the DoE were analyzed with MODDE.

**Table 3:** Shows the individual experiments of the full factorial design of experiments for the screening study

Experiment no.	Temperature °C	pH	CO_2_%
N1	25	7.0	2
N2	25	7.0	10
N3	25	10	2
N4	25	10	10
N5	35	7.0	2
N6	35	7.0	10
N7	35	10	2
N8	35	10	10
N9	30	8.5	6
N10	30	8.5	6
N11	30	8.5	6

**Fig. 2:**
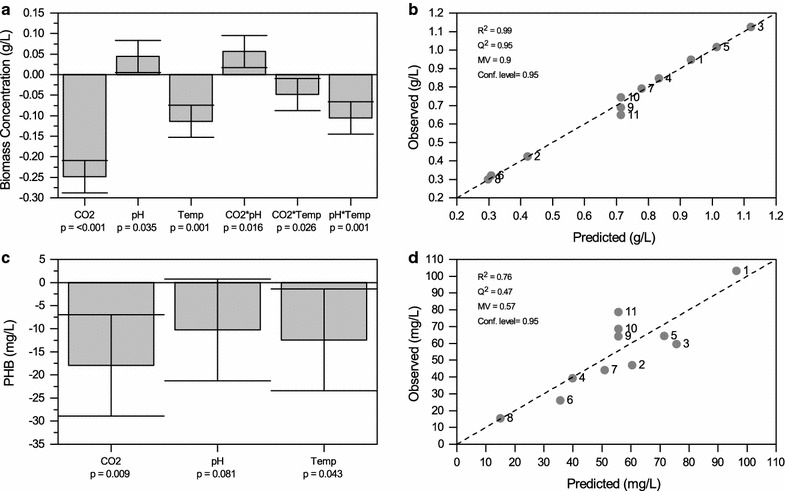
**a**, **c** Represent the coefficient plots of the DoE experiments for *Synechocystis* sp. PCC 6714 under nitrogen and phosphorus limitation, for biomass concentration (**a**) and PHB accumulation (**c**), respectively. **b**, **d** Show the model parameters and observed-versus-predicted plots of the models obtained for biomass concentration (**b**) and PHB accumulation (**d**) under nitrogen and phosphorus limitation

First, the effect of the different factors on biomass formation was determined. As shown in Fig. 2a high CO_2_ concentration significantly reduced the biomass concentration. The model obtained showed that all three factors had a significant influence on the final biomass concentration. While higher CO_2_ concentrations and temperatures decreased biomass formation, higher pH values favored biomass formation. Within the borders of the experimental matrix, highest biomass concentration of 1.12 ± 0.12 g L^−1^ was found at 2% CO_2_, pH 10 and 25 °C.

Significant effects for the interaction terms CO_2_ * pH (increased biomass), CO_2_ * temperature (decreased biomass) and pH * temperature (decreased biomass) have also been obtained from the biomass model.

Next, the effect of the different factors on PHB accumulation was determined. As shown in Fig. 2c PHB accumulation was significantly reduced by increased CO_2_ concentrations, pH values, and temperatures. However, the effect was not significant for the pH value. Within the borders of the experimental matrix, highest PHB accumulation of 103 ± 10 mg L^−1^ (11 ± 1% dcw) was found at 2% CO_2_, pH 7 and 25 °C followed by an average PHB content of 10 ± 1% (dcw) at pH 8.5, 30 °C and 6% CO_2_.

The DoE was done under three different illumination conditions: dark, light and dark/light cycle (16:8 h), respectively. The results obtained showed both dark/light cycle and continuous illumination were suitable for PHB accumulation. No significant difference was observed between the results obtained for maximum PHB accumulation under dark/light cycle (100 ± 10 mg L^−1^, 10.5% dcw) and continuous illumination (103 ± 10 mg L^−1^, 11% dcw). Dark/light cycle was found stimulatory for biomass growth. Maximum biomass concentration obtained under dark/light cycle (1.30 ± 0.1 g L^−1^) was higher when compared to continuous illumination (1.12 ± 0.1 g L^−1^). Cells grown without light showed very little biomass growth (< 0.1 g L^−1^) and PHB accumulation (<1% dcw).

### Photobioreactor cultivation of *Synechocystis* sp. PCC 6714

Based on the results of the design of experiments indicating favorable conditions for both biomass formation and PHB accumulation these two parameters were studied in photobioreactor cultivations. To that end, photoautotrophic cultivation of *Synechocystis* sp. PCC 6714 was established in a 1-L lab scale photobioreactor with the aim to determine whether defined cultivation conditions could improve biomass formation, specific growth rates, and PHB content. In addition, the impact of the nitrogen source on biomass growth was studied. The data from the design of experiments suggested different cultivation conditions for maximum biomass concentration and PHB accumulation under nitrogen and phosphorus limitations. To ensure suitable cultivation conditions for biomass formation and PHB accumulation, four photobioreactor cultivations with different pH set points (7, 8.5, 9 and 10) at 28 °C and 2% CO_2_ were done (data not shown). Highest biomass formation was observed at pH 8.5. Therefore, the following parameters of 2% CO_2_, pH 8.5 and temperature of 28 °C were used for all photobioreactor cultivations.

**Fig. 3:**
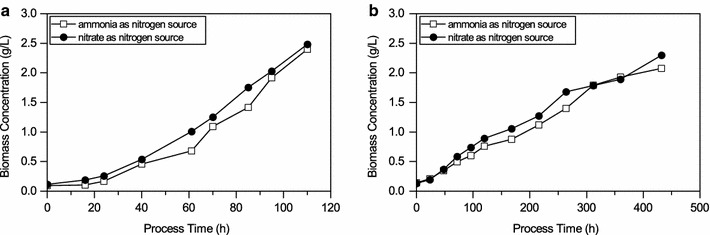
Cultivation of *Synechocystis* sp. PCC 6714 on two different nitrogen sources, nitrate and ammonia, under continuous illumination. Given are biomass concentration over time for **a** photobioreactor cultivations and **b** shake flask experiments. The values shown are the mean of three individual experiments, a single standard deviation is given as *error bar*

Figure 3 shows growth curves obtained for cultivations a) in photobioreactor and b) in shake flasks with either nitrate or ammonia as nitrogen source. The μ_average_ in photobioreactor cultivations was 0.672 ± 0.07 day^−1^ with a µ_max_ of 0.792 ± 0.08 day^−1^ compared to μ_average_ of 0.168 ± 0.02 day^−1^ and µ_max_ of 0.240 ± 0.02 day^−1^ in shake flasks cultivations. This represents a fourfold higher specific growth rate for *Synechocystis* sp. PCC 6714 under defined conditions in the photobioreactor compared to shake flask cultivations.

The obtained results suggest that both nitrogen sources, nitrate and ammonia, are suitable for cultivation of *Synechocystis* sp. PCC 6714 in a photobioreactor and could facilitate biomass growth.

### PHB accumulation in the photobioreactor cultivations

A significant increase of specific growth rate could be successfully shown in photobioreactor runs compared to shake flask experiments. Thus, it was hypothesized that also PHB accumulation is increased in photobioreactor cultivations. Two step cultivations of *Synechocystis* sp. PCC 6714 were done for photoautotrophic production of PHB under nutrient limitations.

**Fig. 4:**
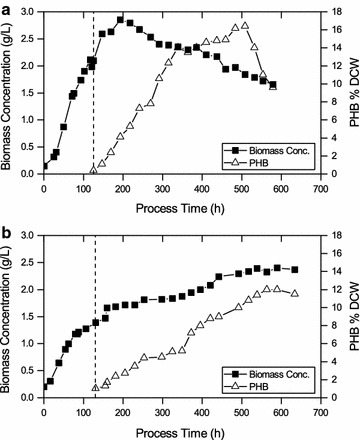
Time course of growth and PHB accumulation under nitrogen and phosphorus limitation for photobioreactor cultivations of *Synechocystis* sp. PCC 6714 for **a** growth on nitrate **b** growth on ammonia. *The *vertical bar* represents the start of the limitation phase. The *values* shown are the mean of three individual experiments)

As shown in Fig. 4a biomass growth occurred in BG-11 media using nitrate as nitrogen source until 120 h with a µ_average_ of 0.38 ± 0.04 day^−1^. Limitation started when cells were harvested and transferred into media without nitrogen and phosphorus. Cell growth ceased after 200 h of cultivation although metabolic activity continued until the end of the process. PHB accumulation started at the early phase of the limitation and a PHB content of 8 ± 1% (dcw) after 6 days of limitation was obtained. PHB accumulated to an intracellular concentration of about 14 ± 1% (dcw) on day 9 and the highest PHB content was observed on day 15 of about 16.4 ± 2% (dcw). A decline in PHB content was detected after day 15. The maximum polymer content per volume of medium was 342 ± 30 mg L^−1^ PHB which corresponds to 14.6 ± 1% (dcw) on day 10 from CO_2_. The highest volumetric PHB production rate obtained was 59 ± 6 mg L^−1^ day^−1^.

Figure 4b shows biomass growth occurred on BG-11 media and ammonia as nitrogen source under a photoautotrophic condition in a lab-scale photobioreactor. Growth occurred until 120 h with a µ_average_ of 0.36 ± 0.04 day^−1^. This value is lower when compared with cultivations with nitrate as a nitrogen source. The intracellular level of PHB at the starting point of the limitation phase was determined to be about 1% (dcw) which is higher than for the cultivation with nitrate. When ammonia was used as nitrogen source for growth, the maximum PHB content obtained under nitrogen and phosphorus limitation was 0.288 ± 0.3 mg L^−1^ PHB on day 19 which corresponds to 14 ± 1% (dcw) PHB. The highest volumetric PHB production rate with ammonia as nitrogen source was 51 ± 5 mg L^−1^ day^−1^.

### Elemental analysis of macronutrients from photobioreactor samples

**Fig. 5:**
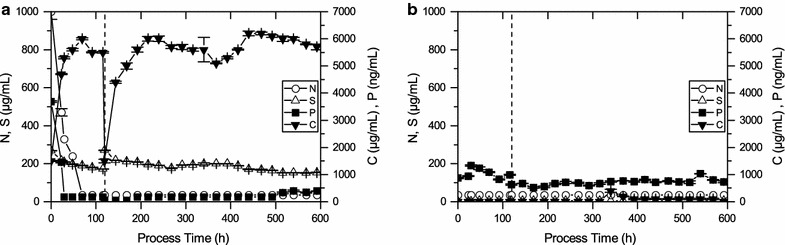
Time course of residual macronutrient concentrations for nitrogen, phosphorus, sulfur, and carbon in photobioreactor cultivations of *Synechocystis* sp. PCC 6714 cultivated photoautotrophically and with nitrate as the nitrogen source is shown **a** in the culture supernatant **b** intracellular concentration of the macronutrients. *The *vertical bar* represents the start of the limitation phase. The *values* shown are the mean of three individual experiments, a single standard deviation is given as *error bar*

The concentrations of macronutrients nitrogen, phosphorus, sulfur and carbon were characterized in photobioreactor cultivations of *Synechocystis* sp. PCC 6714 under photoautotrophic growth conditions with nitrate as the nitrogen source. Elemental analysis of the culture supernatant showed uptake of nitrogen and phosphorus during the first 50 h of the growth phase (0–120 h) (Fig. 5a). Nitrogen concentrations reduced to below the detection limit (70 mg L^−1^) during the growth phase and remained below the detection limit until the end of the cultivation. Accumulation of carbon up to 6 ± 0.5 g L^−1^ in the culture supernatant was observed during both, the growth and limitation phase whereas no such accumulation of carbon was observed intracellularly. Uptake of small amounts of sulfur was detected both during growth and limitation phase. Figure 5b shows that the intracellular concentration of phosphorus increase during the first 25 h of the growth phase (0–120 h) and subsequently decreased until the limitation phase was started. During the limitation phase, the intracellular level of phosphorus remained almost constant. The intracellular concentration of carbon, nitrogen, and sulfur during both growth and limitation phase was below the detection limits.

### Size determination of PHB granules

**Fig. 6:**
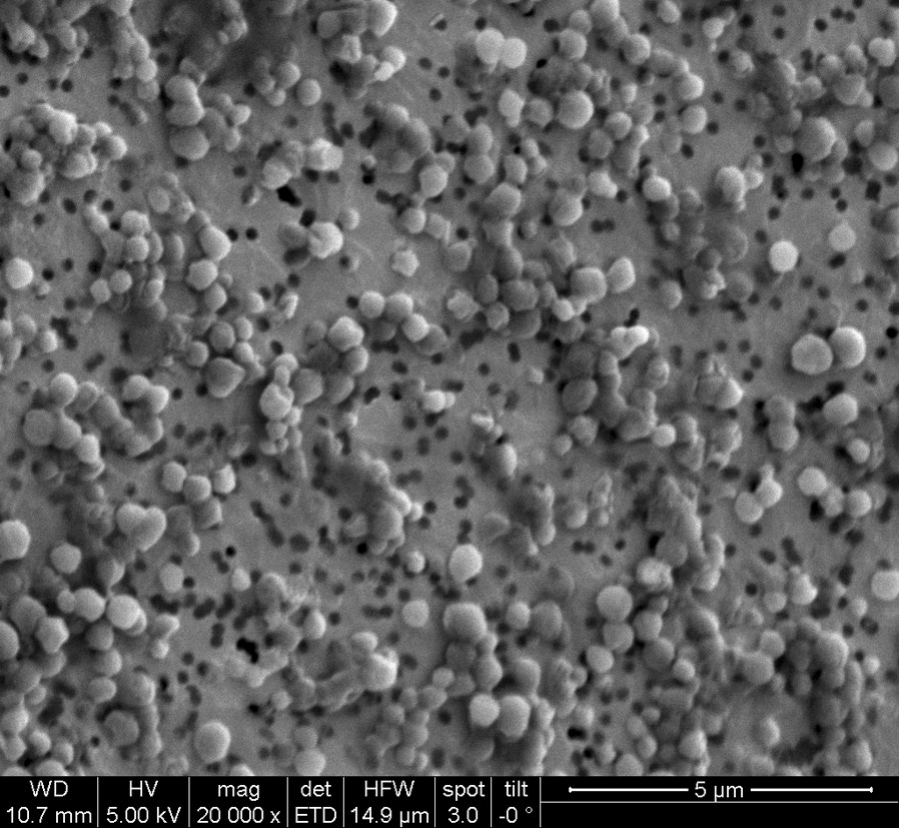
Scanning electron microscopy image of PHB granules from *Synechocystis* sp. PCC 6714 cells cultivated photoautotrophically and N and P limitation

Apart from quantitative determination of the PHB content, it was of interest to visualize PHB granules in this strain. To this end, scanning electron microscopy (SEM) was used to determine shape and size of the PHB granules extracted from *Synechocystis* sp. PCC 6714. Figure 6 shows a SEM image of PHB granules from the photoautotrophic cultivation of nitrogen and phosphorus limited cells. Image analysis of PHB granule using ImageJ plugin Fiji showed electron transparent particles with average diameter of 0.7 µm and maximum size of 0.95 µm. Cells cultured in complete BG-11 media were used as a control. They were homogenized and prepared the same way for analysis by scanning electron microscopy. For the unlimited control cells, no electron transparent particles were observed.

## Discussion

### Initial characterization

Initial strain characterization showed the flexibility of *Synechocystis* sp. PCC 6714 for utilization of different carbon sources for growth. Although growth on carbonate and acetate occurred with more or less the same µ_average_ 0.095 ± 0.01 day^−1^ for growth on carbonate (1.45 ± 0.1 g L^−1^) higher biomass concentration was detected when compared to acetate (1.25 ± 0.1 g L^−1^). This is in contrast to results which were shown for other cyanobacteria where acetate resulted in higher biomass concentrations (De Philippis et al. 1992). As it was expected, growth on glucose occurred at higher µ_average_ (0.107 ± 0.01 day^−1^) and the observation was in line with previous reports of Vermaas (1996) and Wang et al. (2002) where higher growth rates were detected for *Synechocystis* sp. PCC 6803 when glucose was used as the substrate.

### PHB formation under nutrient limitations

In this study, photoautotrophic PHB accumulation in *Synechocystis* sp. PCC 6714 has been explored for the first time. Under photoautotrophic conditions and nitrogen limitation, a rise in PHB pool up to 10 ± 1% (dcw) was observed. Stimulation of PHB accumulation by nitrogen limitation has been previously explored by Lee et al. (1995) and (2005) where an increase in PHB accumulation under nitrogen limitation was ascribed to high intracellular concentrations of NADPH. Supplementation of acetate under nitrogen deficiency was found to be stimulatory for PHB accumulation in *Synechocystis* sp. PCC 6714. This has also been observed for other cyanobacteria. Wu et al. (2002) reported 15 ± 1% (dcw) PHB accumulation for *Synechocystis* sp. PCC 6803 when acetate was supplemented. Increase in PHB production by acetate supplementation has been explained by Dawes (1992) as the result of direct utilization of the substrate for polymer synthesis. Simultaneous limitations of nitrogen and phosphorus under photoautotrophy appeared to be a suitable stimulant for PHB accumulation since up to 16.4 ± 2% (dcw) PHB were detected under these conditions. These results are in line with earlier findings of Pal et al. (1998) and Nishioka et al. (2001) where it was shown that PHB accumulation could be increased by phosphate unavailability for *Azotobacter chroococcum* and *Synechococcus* sp. MA-19, respectively.

Limitation of gas exchange under nitrogen and phosphorus deficiency in *Synechocystis* sp. PCC 6714 reduced the intracellular PHB pool and biomass concentration significantly. This could be due to an inhibitory effect of oxygen caused by a build-up in the culture vessel. Oxygen competes with CO_2_ during photorespiration for ribulose-1, 5-bisphosphate carboxylase/oxygenase (RuBisCO) enzyme to convert CO_2_ to biomass (Raso et al. 2012). The findings from this study are in contrast with the results reported by Panda and Mallick (2007) which showed GEL can significantly boost PHB content in *Synechocystis* sp. PCC 6803. The different results of the two studies could be due to the different conditions used for the experiments.

### Optimization of cultivation parameters

Key parameters influencing growth and PHB content in *Synechocystis* sp. PCC 6714 were identified using a multivariate design of experiments. It was found that biomass formation and PHB accumulation were a function of various cultivation parameters. Within the borders of the specified experimental matrix, the highest biomass concentration (1.12 ± 0.1 g L^−1^) and highest PHB accumulation (11 ± 1% dcw) were obtained at 2% CO_2_ concentration. This observation, however, is in contrast to earlier findings of Eberly and Ely (2012) where growth rates and accumulation of carbon storage compounds were enhanced with increased CO_2_ concentrations in the thermophilic cyanobacterium *Thermosynechococcus elongatus*. This difference in growth behavior might be due to lower CO_2_ solubility at 50 °C used for *T. elongates* compared to 28 °C used for *Synechocystis* sp. PCC 6714. Additionally, a temperature of 28 °C used for photobioreactor cultivations of *Synechocystis* sp. PCC 6714 was favorable for PHB accumulation. This is in line with previous observations of Panda et al. (2006) for *Synechocystis* sp. PCC 6803 where the temperature range of 28–32 °C was preferred for PHB accumulation.

Cultivations with either continuous illumination or dark/light cycles did not show a significant difference in PHB content. However, cells grown without light showed very little biomass growth and PHB accumulation.

### Cultivation of cyanobacteria in the photobioreactor

Interestingly, photobioreactor cultivations of *Synechocystis* sp. PCC 6714 showed fourfold higher average specific growth rate when compared to shake flask cultivations. This observation is likely due to well-defined conditions in the photobioreactor compared to uncontrolled shake flask cultivations. It has been shown that under controlled conditions, growth rates and biomass concentration can be improved in microalgae cultivations (García-Malea et al. 2009; Ugwu et al. 2008). This underlines the importance of defined conditions to obtain highly productive processes (Pruvost et al. 2011). It has been shown biomass and lipid productivity in microalgae can be improved significantly under defined conditions of a photobioreactor.

It was observed that for photobioreactor cultivations using ammonia as the nitrogen source during the growth phase, *Synechocystis* sp. PCC 6714 already showed a PHB content of 1% (dcw) at the end of the growth phase. This suggests that the cells might have already been limited during the growth phase. One explanation for this phenomenon might be that the uptake of ammonia is affected by the pH-dependent equilibrium between NH_3_ and NH_4_^+^ and therefore might not be sufficiently available to the cells throughout the growth phase. Furthermore, it would be expected that uptake rates for ammonia are higher than for nitrate since ammonia can directly be utilized by the cell, whereas nitrate needs to be converted into ammonia first. Therefore, the energetic cost for the cell to take up ammonia is lower compared to nitrate (Dortch and Postel 1989; Syrett 1981). In the present study, higher growth rates and PHB contents were observed for cultivations with nitrate as nitrogen source compared to cultivations with ammonia as nitrogen source. This has been previously reported by Rückert and Giani (2004) where higher cell densities and a higher protein content was detected in *Microcystis viridis* when nitrate was used instead of ammonia or urea. In addition, high concentrations of ammonia have been shown to be toxic to some cyanobacteria (Drath et al. 2008), while no such toxicity has been reported for nitrate. Problems related to solubility and toxicity of ammonia could be overcome by implementing a continuous feeding strategy.

### Potential of *Synechocystis* sp. PCC 6714 for photoautotrophic production of PHB

16.4% (dcw) PHB were obtained for *Synechocystis* sp. PCC 6714 from CO_2_. PHB content of 11–13% and 29% (dcw) are reported, respectively, in wild-type and genetically modified *Synechocystis* sp. PCC 6803 by Klotz et al. (2016) and Khetkorn et al. (2016). Also 27 and 23% (dcw) for *Synechococcus* sp. MA-19 and *Nostoc muscorum*, respectively, have been obtained (Nishioka et al. 2001; Sharma and Mallick 2005). However, total product concentration and volumetric rates for these strains have not been described and therefore it is challenging to evaluate the mentioned strains in terms of PHB productivity only based on the reported percentage of dry cell weights. In this respect, Wu et al. in (2001) reported total PHB concentration of 16 to 27 mg L^−1^ PHB for *Synechocystis* sp. PCC 6803. For C*alothrix scytonemicola* TISTR 8095 PHB content of about 25.4% (dcw) was reported with a total PHB concentration of 356 mg L^−1^ after 60 days of cultivation (Kaewbai-Ngam et al. 2016). In this study, the maximum polymer content per volume of the medium of 342 ± 34 mg L^−1^ PHB was observed after 10 days of limitation which corresponds to 14.6 ± 1% (dcw) PHB. The highest volumetric production rate of PHB observed was 59 ± 6 mg L^−1^ day^−1^. Available literature so far has not used this parameter, therefore our obtained value cannot be compared to other studies. Additional accumulation of a carbon-containing compound (up to 6 g L^−1^ of carbon) in the culture supernatant during photobioreactor cultivations of *Synechocystis* sp. PCC 6714 could be due to the formation of extracellular polysaccharides as previously reported by De Philippis and Vincenzini (1998). However, this observation requires further investigation.

In this study, the cyanobacterium *Synechocystis* sp. PCC 6714 has been reported as PHB producer for the first time. The results suggest *Synechocystis* sp. PCC 6714 to be suitable as a potential host strain for photoautotrophic PHB production. The specific growth rate, total PHB content and volumetric PHB production rate were increased significantly only by controlling process conditions in two-step batch cultivations in a photobioreactor. In order to obtain a robust process further optimization with respect to a suitable process strategy supporting both biomass formation as well as stable PHB production is required. A potential strategy that shall be further investigated is to optimize the media to provide nitrogen and phosphorus in a way that enables a one-step process without the need to exchange the media.

## Abbreviations

PHB: polyhydroxybutyrate
dcw: dry cell weight
SEM: scanning electron microscopy
SED: scanning electron detector
µ_average_: average specific growth rate
µ_max_: maximum specific growth rate
PAR: photosynthetically active radiation
OD_750_: optical density at 750 nm
GEL: gas exchange limitation
DoE: design of experiment
MLR: multiple linear regression
MV: model validity
R^2^: coefficient of determination
Q^2^: predictability of a model
RP: reproducibility of a model
ICP-OES: inductively coupled plasma-optical spectroscopy
Ir: internal standard
RuBisCO: ribulose-1, 5-bisphosphate carboxylase/oxygenase
